# Dilated Cardiomyopathy: A Paradigm of Revolution in Medicine

**DOI:** 10.3390/jcm9113385

**Published:** 2020-10-22

**Authors:** Marco Merlo, Antonio Cannatà, Gianfranco Sinagra

**Affiliations:** 1 Cardiovascular Department, Azienda Sanitaria Universitaria Giuliano Isontina (ASUGI), University of Trieste, 34100 Trieste, Italy; anto.cannata@gmail.com (A.C.); gianfranco.sinagra@asugi.sanita.fvg.it (G.S.); 2 James Black Centre, School of Cardiovascular Medicine and Sciences, King’s College London British Heart Foundation Centre of Excellence, 125 Coldharbour Lane, London SE5 9NU, UK

Dilated Cardiomyopathy (DCM) has a straightforward and apparently “simple” definition: a heart muscle disease characterized by left ventricular (LV) or biventricular dilation and systolic dysfunction in the absence of either pressure or volume overload or coronary artery disease sufficient enough to explain the dysfunction [1]. DCM currently carries a relatively benign outcome, significantly improved with respect to the past decades. Contemporary analysis shows the survival/free from heart transplant rate beyond 85% at 10-year follow-up [2]. Nevertheless, the knowledge regarding pathophysiology, aetiology, diagnostic workup and prognostic stratification of DCM is rapidly and progressively evolving, reflecting the clinical management of the disease that remains extremely challenging in daily practice [3]. Indeed, DCM patients are often relatively young at the time of diagnosis (between their 30s and 50s) with a low-co-morbidity profile, and their current diagnostic workup and risk stratification is characterized by several pitfalls (particularly regarding the arrhythmic risk). Consequently, a not-negligible proportion of DCM patients still experience an unfavourable prognosis, particularly in the short-term [2].

One of the reasons behind this complicated scenario is the heterogeneous aetiology of the disease. DCM is an “umbrella” term describing the final common pathway of different pathogenic processes and gene–environment interactions. More commonly than once believed, DCM recognizes a complex genetic background, far from being a monogenic disease, with multiple unknown epigenetic interactions. On the other side, it might be the result of possible extrinsic triggers (i.e., tachyarrhythmias, hypertension, alcohol, chemotherapy, inflammation), which, once removed, promote a reverse remodelling. Therefore, the term “idiopathic” DCM is progressively vanishing, and investigations on the complex interaction between environmental factors and genetic background are increasing. Future research in this perspective is likely to result in better prognostic stratification and ultimately targeted therapy [4].

Noteworthily, thorough phenotyping (through modern imaging techniques such as speckle tracking echocardiography or tissue characterization by cardiac magnetic resonance) and genotyping of DCM patients represent the basis for their optimal clinical management. Furthermore, compelling evidence shows that DCM is not exclusively a cardiological disease, requiring a multidisciplinary team (including geneticist, neurologist, radiologist and other specialists) for accurate management. Therefore, a novel approach to DCM patients, including comprehensive evaluation, should be promoted to tailor therapeutic strategies in the era of precision medicine.

Starting from these concepts, the idea of this Special Issue is to explore the DCM universe providing updated knowledge on pathophysiology, future directions of the research on DCM and practical guidelines useful for clinical management of DCM patients.

A series of focused reviews, meta-analyses and original articles are reported in this Special Issue with the precise aim of providing a deep insight into crucial gaps of knowledge in DCM. In particular, it extensively discusses the pathophysiology, mechanisms underlying the disease and the interaction between genetic background, molecular pathways and environmental triggers, as the basis for future targeted therapies [5,6,7]. The knowledge of precise genetic pathogenesis and molecular mechanisms causing DCM has stimulated the research towards new treatments targeting gene expression [8,9]. Shifting from symptomatic treatments to targeted therapy on specific disease mechanisms represents the new mindset from slowing disease progression to disease reversal. Furthermore, some articles present in this Special Issue explore the genetic background of DCM, such as mutations in *DES, LMN* and *TTN,* remarking once again the current cultural revolution in this field of medicine. In the future, we might indeed abandon the current general definition of DCM, switching towards specific diseases such as Desminopathy, Laminopathy or Titinopathy and so on, each of them with specific diagnostic workup, prognostic stratification and therapeutic strategies [10,11,12]. Importantly, the need of a multidisciplinary network involving different specialists clearly emerges in specific and challenging diseases, such as Duchenne-related DCM in order to improve the global clinical management of those challenging patients [13]. Finally, the prognostic stratification of DCM has been further explored, focusing on (1) the identification of specific subgroups of DCM without a structural myocardial disease, such as the tachycardia-induced cardiomyopathy [14]; (2) the application of gender medicine to DCM clinical management [15]; (3) the usefulness of tissue characterization by cardiac magnetic resonance in the multi-parametric approach od DCM patients [16] and (4) the role of the left atrium, other than just the left ventricle, as a therapeutic target of pharmacological and non-pharmacological treatments in DCM [17]. Finally, a section is dedicated to the characterization of left ventricular non-compaction that is frequently encountered in clinical practice in overlap with the DCM phenotype [16,18]. The definition of left ventricular non-compaction as a specific cardiomyopathy or, more likely, as a specific trait of genetic cardiomyopathy is debated, and it still represents a gap in knowledge in clinical management, particularly for the first phases of the disease.

Far from providing the absolute truth, this Special Issue is intended to help physicians (not only cardiologists) in their everyday clinical practice to deal with patients affected by DCM in a multifaceted, multidisciplinary and individualized approach.
